# Comparative Effects Between Direct Oral Anticoagulants for Acute Venous Thromboembolism: Indirect Comparison From Randomized Controlled Trials

**DOI:** 10.3389/fmed.2020.00280

**Published:** 2020-06-19

**Authors:** Guowei Li, Jie Zeng, Junguo Zhang, Lehana Thabane

**Affiliations:** ^1^Center for Clinical Epidemiology and Methodology (CCEM), Guangdong Second Provincial General Hospital, Guangzhou, China; ^2^Department of Health Research Methods, Evidence, and Impact (HEI), McMaster University, Hamilton, ON, Canada; ^3^Biostatistics Unit, St. Joseph Healthcare-Hamilton, Hamilton, ON, Canada

**Keywords:** venous thromboembolism, direct oral anticoagulant, major bleeding, comparative effect, efficacy, safety

## Abstract

**Background:** There is no direct comparison from clinical trials amongst the direct oral anticoagulants (DOACs) in patients with acute venous thromboembolism (VTE), leaving an evidence gap in decision-making regarding the choice of a DOAC.

**Methods:** We performed a systematic review for an indirect comparison from randomized controlled trials (RCTs) for comparative effects amongst DOACs in the patients with acute VTE.

**Results:** A total of 16 RCTs were included for analyses, among which three for dabigatran (*n* = 7,963 patients), six rivaroxaban (*n* = 17,935), five apixaban (*n* = 12,823), and two edoxaban (*n* = 9,286). There was no significant difference in risk of recurrent VTE (evidence quality: low) and major bleeding (evidence quality: very low) for treatment effects between the four DOACs. Albeit non-significantly, apixaban seemed to have a lowest risk of major bleeding while rivaroxaban had a smallest risk of VTE. Although in general all the included trials were comparable, data from the included trials indicated that the assumption of transitivity may be challenged. Further methodological research including simulation studies, using a net-benefit or benefit-harm approach, running ranking probability analysis, and developing decision aids with machine-learning may be a worthwhile endeavor to help with the choice of DOACs in patients with acute VTE.

**Conclusions:** To conclude, based on results from the indirect comparison no significant difference in the efficacy and safety was found among the DOACs in patients with acute VTE. More evidence from direct comparative trials is needed to further inform the choice of DOACs in patients with acute VTE.

## Introduction

Venous thromboembolism (VTE), including pulmonary embolism (PE) and deep vein thrombosis (DVT), is the third most common cause of vascular death following myocardial infarction and stroke (1). To treat patients with acute VTE, current guidelines recommend using a parenteral anticoagulant for at least 5 days and subsequently an oral vitamin K antagonist (VKA) for at least 3 months (2, 3). Nevertheless, concerns are raised about the use of parenteral anticoagulant due to its inconvenient administrations and the use of VKA because of its close laboratory monitoring.

Direct oral anticoagulants (DOACs), including the direct thrombin inhibitor (dabigatran) and factor Xa inhibitors (apixaban, rivaroxaban, and edoxaban), have been recommended to treat acute VTE in the current guidelines, given their promising benefit-harm profiles, more predictable pharmacodynamics effects, less food and drug interactions, and less need for coagulation monitoring (4, 5). However, there is no direct comparison from clinical trials amongst the four DOACs, leaving an evidence gap in decision-making regarding the choice of a DOAC. In this study, we aimed to conduct a systematic review to perform an indirect comparison from randomized controlled trials (RCTs) for comparative effects amongst DOACs in the patients with acute VTE. We also appraised advantages and disadvantages of the indirect comparison technique, and provided methodological insights into the potential clinical implications based on the findings.

## Methods

We conducted this systematic review based on guidance from the Cochrane Handbook of Systematic Reviews and reported results according to PRISMA (the Preferred Reporting Items for Systematic Reviews and Meta-analyses) (6, 7).

### Search Strategy and Study Selection

We searched MEDLINE, CINAHL, CENTRAL, and EMBASE up to November 30th, 2019 to retrieve eligible RCTs. We used descriptors including synonyms for trials, DOACs, and VTE in various combinations for the search process (Supplemental Table 1 shows the terms used in the search). The website www.clinicaltrials.gov was also searched for potential unpublished and ongoing studies (up to December 8th, 2019).

RCTs assessing efficacy and safety of DOACs vs. LMWH (low molecular weight heparin), UFH (unfractionated heparin), or VKA for treatment of acute VTE in adults were eligible for inclusion, where an acute VTE referred to a period within the first six months from the index VTE that had an objectively confirmed diagnosis (3). If patients in a same trial were reported in multiple publications or at different time points, only the study with the largest sample size and longest follow-up was included. We excluded those trials that compared DOACs vs. heparin or VKA to treat acute VTE but did not focus on the efficacy or safety profiles. We also excluded those studies without sufficient information for data extraction. Ongoing studies were included if the authors contacted by us could provide detailed information for our analyses.

### Outcomes

Primary outcomes included the efficacy outcome (recurrent symptomatic VTE including new episode of DVT or PE), and the safety outcome (composite of major bleeding). Secondary outcomes were clinically relevant non-major bleeding (CRNB) and all-cause death.

### Data Extraction

Two reviewers (GL and JZ) screened and chose studies for inclusion independently. We used the kappa statistics to quantify the agreement between the two reviewers (8, 9). A third reviewer (LT) was resorted if disagreement between the two reviewers could not be resolved by discussion. Two reviewers (GL and JZ) independently extracted data from the included RCTs on patient characteristics, intervention details, control groups, outcome measurement, study time, and study design information including sequence generation, allocation, blinding method, and type of trial design (parallel, cross-over, or factorial).

We used the Risk of Bias evaluation tool from Cochrane Collaboration to assess the quality of individual included RCTs (6). The Risk of Bias tool included sequence generation, allocation concealment, blinding, outcome data completeness, outcome reporting, and other issues.

### Statistical Analyses

Due to no head-to-head comparisons from RCTs amongst DOACs, we performed an adjusted indirect comparison using a common comparator based on the Bucher technique (10, 11). We calculated the pooled hazard ratios (HRs) with their corresponding 95% confidence intervals (CIs) for comparative efficacy and safety amongst DOACs. We used the highest dose of the DOACs for comparisons if more than one dose was reported in the included RCTs. If more than one trial investigating a same DOAC, we first ran a random-effects model to pool the trials before performing the indirect comparison.

Two predefined subgroup analyses were conducted by type of VTE (DVT and PE) and type of controls (VKA, UFH, and LMWH). If a conventional meta-analysis was performed before the indirect comparison, we carried out three pre-specified sensitivity analyses (1) by using the fixed-effects model, (2) by excluding the high-risk-of-bias trials from the meta-analysis, and (3) by excluding the trials in which the percentages of patients with cancer were over 10%.

### Assessment of Transitivity

We assessed the transitivity of the included trials by comparing their patients, interventions, controls, and outcomes (12). We compared the distributions of potential effect modifiers across the different comparisons including age, gender, comorbidity, dosage, time in therapeutic range (TTR), setting, outcome definition and adjudication, and study time duration (13).

### Assessment of Certainty of Evidence

We used the Grading of Recommendations, Assessment, Development, and Evaluation (GRADE) approach to rate the certainty in evidence for primary outcomes (14, 15). We first rated the certainty of evidence in direct comparison (DOACs vs. non-DOACs) according to risk of bias, directness of evidence, impression, inconsistency, and publication bias. Indirect comparison was subsequently rated based on results from rating of direct comparison and assessment of transitivity in the included trials (16).

## Results

**Figure 1:**
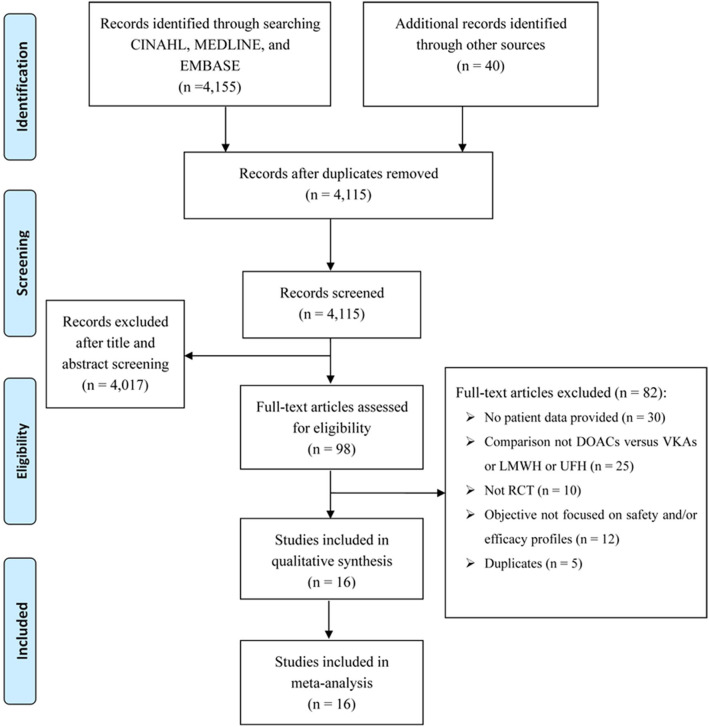
Study flow diagram showing the study selection process.

**Figure 2:**
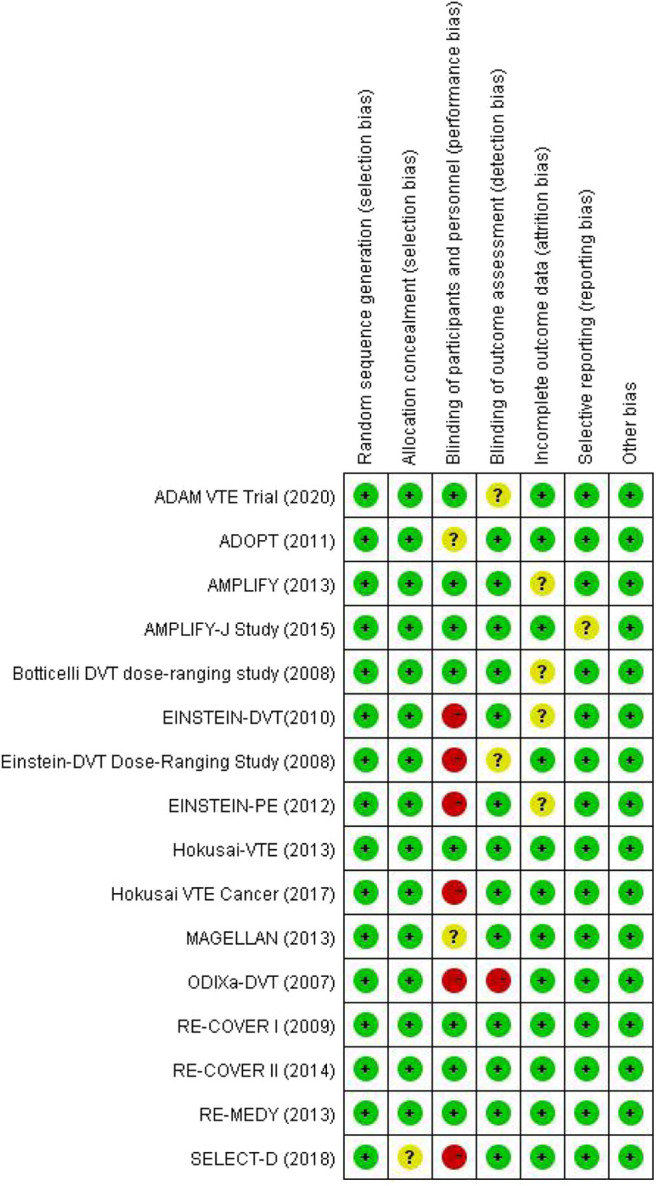
Assessment of risk of bias for the included trials.

There were 4,155 records identified for screening. A total of 16 studies were included for analyses, among which three for dabigatran (17–19), six rivaroxaban (20–25), five apixaban (26–30), and two edoxaban (31, 32) (Figure 1 shows the flow diagram for study selection). The numbers of participants were 7,963 (40% for females) in RCTs assessing dabigatran, 17,935 (44% females) for rivaroxaban, 12,823 (46% females) for apixaban, and 9,286 (44% females) for edoxaban, respectively. Table 1 displays the characteristics of participants in the included trials. The average age ranged from 54 to 71 years. Three trials included patients with cancer exclusively; i.e., the percentages of patients with cancer were 100% in SELECT-D (23), ADAM VTE (28), and Hokusai VTE Cancer (32). The percentages of patients with creatinine clearance < 50 ml/min ranged from 5 to 22%. Results of the quality assessment for individual trials are presented in Figure 2. Six trials were rated high risk of bias regarding the domain of blinding.

**Table 1:** Description of characteristics for included trials.

**Study name (year) [reference number]**	**Population characteristics**							**Information on Intervention/Control (dosage, administration, duration)**	**Outcome measure**		**Follow-up period (months)**
	**Number of randomized patients**	**Study arm**	**Sample size for each arm (% for females)**	**Age: years**	**% for patientswith cancer**	**Time in therapeutic range**	**% for patient with creatinine clearance <50 ml/min**		**Efficacy**	**Safety**	
**DABIGATRAN (*****n*** **= 3)**											
RE-COVER I (2009) (15)	2,539	DOAC	1,273 (42%)	55	5%	NA	5.0%	Dabigatran (150 mg orally twice daily for 6 months)	Venous thromboembolism or related death	Major bleeding event or clinically relevant bleeding	6
		VKA	1,266 (41%)	54	4.5%	60.0%	4.5%	Warfarin (150 mg dose-adjusted for 6 months)			
RE-MEDY (2013) (17)	2,856	DOAC	1,430 (39%)	55	4.2%	NA	NA	Dabigatran (150 mg orally twice daily for 6 months)	Recurrent symptomatic VTE or VTE mortality	Major bleeding event or clinically relevant bleeding	36
		VKA	1,426 (39%)	54	4.1%	65.3%	NA	Warfarin (at a fixed dose of 150 mg twice daily for 6 months)			
RE-COVER II (2014) (16)	2,568	DOAC	1,280 (39%)	56	3.9%	NA	NA	Dabigatran (150 mg twice daily for 6 months)	Recurrent symptomatic, confirmed VTE and related deaths	Major bleeding	6
		VKA	1,288 (40%)	57	3.9%	56.9%	NA	Warfarin (150 mg for 6 months)			
**RIVAROXABAN (*****n*** **= 6)**											
ODIXa-DVT (2007) (22)	604	DOAC	119 (41%)	58.5	NA	NA	NA	Rivaroxaban (10 mg twice daily for 12 weeks)	Proximal deep vein thrombosis, symptomatic PE, or VTE-related death	Major bleeding	3
			117 (43%)	57.5	NA	NA	NA	Rivaroxaban (20 mg twice daily for 12 weeks)			
			121 (35%)	61.4	NA	NA	NA	Rivaroxaban (30 mg twice daily for 12 weeks)			
			121 (38%)	59.5	NA	NA	NA	Rivaroxaban (40 mg twice daily for 12 weeks)			
		LMWH+VKA	126 (38.9%)	58.4	NA	NA	NA	Enoxaparin 1 mg/kg BID followed by VKA for 12 weeks			
Einstein-DVT Dose-Ranging Study (2008) (23)	543	DOAC	135 (53%)	58	8%	NA	NA	Rivaroxaban (20 mg once daily for 84 days)	Composite of symptomatic venous thromboembolism and asymptomatic deterioration in thrombotic burden	Major bleeding and clinically relevant nonmajor bleeding	3
			134 (49%)	57	10%	NA	NA	Rivaroxaban (30 mg once daily for 84 days)			
			136 (48%)	60	12%	NA	NA	Rivaroxaban (40 mg once daily for 84 days)			
		LMWH+VKA	137(47%)	57	7%	NA	NA	Combination of LMWH and VKA			
EINSTEIN-DVT (2010) (18)	3,449	DOAC	1,731 (43%)	56	6.8%	NA	6.9%	Rivaroxaban (15 mg twice daily for 3 weeks, then 20 mg daily for 3, 6, or 12 months)	Symptomatic recurrent VTE	Clinically relevant bleeding	12
		LMWH+VKA	1,718 (44%)	56	5.2%	57.7%	7.5%	Enoxaparin followed by a warfarin or acenocoumarol for 3, 6, or 12 months			
EINSTEIN-PE (2012) (19)	4,832	DOAC	2,419 (46%)	58	4.7%	NA	8.8%	Rivaroxaban (15 mg twice daily for 3 week, then 20 mg daily for 3, 6, or 12 months)	Symptomatic recurrent VTE	Clinically relevant bleeding	12
		LMWH+VKA	2,413 (48%)	58	4.5%	62.7%	8.0%	Enoxaparin followed by an adjusted-dose VKA for 3, 6, or 12 months			
MAGELLAN (2013) (20)	8,101	DOAC	4,050 (44%)	71	7.3%	NA	21.5%	Rivaroxaban (10 mg once daily for 35 days)	Composite of asymptomatic proximal or symptomatic VTE	Composite of major or clinically relevant nonmajor bleeding	3
		LMWH	4,051 (46%)	71	7.3%	NA	21.5%	Enoxaparin (subcutaneously 40 mg once daily for 10 days)			
SELECT-D (2018) (21)	406	DOAC	203 (43%)	67	100%	NA	NA	Rivaroxaban (15 mg twice daily for 3 weeks, then 20 mg once daily for 6 months)	Symptomatic recurrent VTE	Major bleeding and clinically relevant non-major bleeding	24
		UFH	203 (52%)	67	100%	NA	NA	Dalteparin (200 IU/kg daily during month 1, then 150 IU/kg daily for months 2–6)			
**APIXABAN (*****n*** **= 5)**											
Botticelli DVT dose-ranging study (2008) (28)	520	DOAC	130 (36%)	56	8.5	NA	NA	Apixaban (5 mg twice-daily for 84–91 days)	Symptomatic recurrent VTE and asymptomatic deterioration in thrombotic burden	Composite of major and clinically relevant, non-major bleeding	3
			134 (43%)	59	4.5	NA	NA	Apixaban (10 mg twice-daily for 84–91 days)			
			128 (35%)	60	7.0	NA	NA	Apixaban (20 mg once-daily for 84–91 days)			
		LMWH+VKA	128 (36%)	59	8.6%	NA	NA	LMWH followed by a VKA for 3 months			
ADOPT (2011) (25)	6,528	DOAC	3,255 (50%)	67	9.6%	NA	NA	Apixaban (2.5 mg twice daily for 30 days)	Composite of death related to VTE, PE, symptomatic DVT, or asymptomatic proximal-leg DVT	Major bleeding or clinically relevant bleeding	3
		LMWH	3,273 (52%)	67	9.8%	NA	NA	Enoxaparin (subcutaneously 40 mg once daily for 6 to 14 days)			
AMPLIFY (2013) (24)	5,395	DOAC	2,691 (42%)	57	2.5%	NA	6.5%	Apixaban (10 mg, twice daily for 7 days, then 5 mg twice daily for 6 months)	Recurrent symptomatic VTE or VTE mortality	Major bleeding	6
		VKA	2,704 (41%)	57	2.8%	NA	6.1%	Enoxaparin, followed by warfarin for 6 months			
AMPLIFY-J Study (2015) (27)	80	DOAC	40 (45%)	64.3	NA	NA	5.0%	Apixaban was initiated at 10 mg twice daily for 7 days, followed by 5 mg twice daily for 23 weeks	Recurrent symptomatic VTE or VTE-related death	Major bleeding	8
		UFH	40 (57.5%)	66.1	NA	70.1%	20.0%	Unfractionated heparin (UFH) and warfarin for 24 weeks			
ADAM VTE Trial (2020) (26)	300	DOAC	150 (52%)	64.4	100%	NA	9.3%	Apixaban (10 mg twice daily for 7 days followed by 5 mg twice daily for 6 months)	Recurrent VTE or arterial thromboembolism	Major bleeding	6
		UFH	150 (51.3%)	64.0	100%	NA	9.3%	Dalteparin (200 IU/Kg daily for 30 days followed by 150 IU/kg for months 2 through 6)			
**EDOXABAN (*****n*** **= 2)**											
Hokusai-VTE (2013) (29)	8,240	DOAC	4,118 (43%)	56	9.2%	NA	6.5%	Edoxaban (30 or 60 mg once daily for 3 to 12 months)	Symptomatic recurrent VTE	Clinically relevant bleeding	12
		VKA	4,122 (43%)	56	9.5%	63.5%	6.6%	Warfarin for 12 months			
Hokusai VTE Cancer (2018) (30)	1,046	DOAC	522 (47%)	64	100%	NA	7.3% (30–50 mL/min)	Edoxaban (60 mg once daily for 3 to 12 months)	Symptomatic recurrent VTE	Major bleeding	12
		UFH	524 (50%)	64	100%	NA	6.5% (30–50 mL/min)	Dalteparin at a dose of 200 IU/kg for 3 to 12 months			

**Figure 3:**
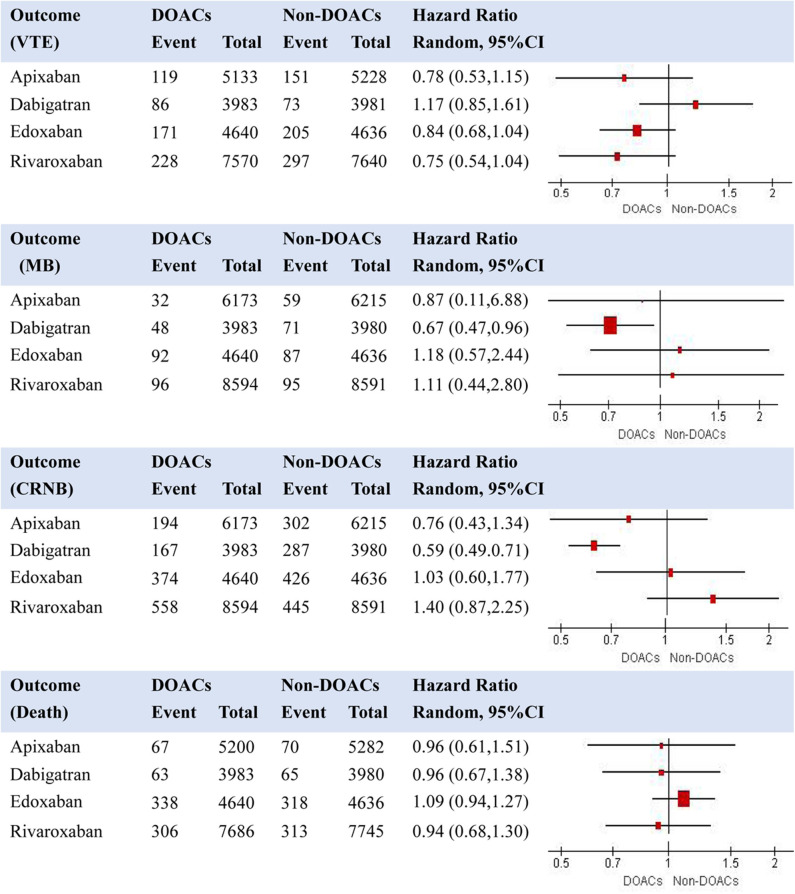
Effects of individual DOACs compared with non-DOACs on risk of recurrent VTE, MB, CRNB, and death (note: VTE, venous thromboembolism; MB, major bleeding; CRNB, clinically relevant non-major bleeding).

We first performed random-effects pooled analyses for the effects of different DOACs compared with their controls (Supplemental Figure 1 presents findings from the individual meta-analyses). Results are shown in Figure 3 regarding the effects of DOACs compared with non-DOACs on VTE, major bleeding, CRNB, and death. When compared with non-DOACs, a smallest HR for risk of VTE was observed in the pooled analysis for rivaroxaban (HR = 0.75, 95% CI: 0.54–1.04), while a smallest HR for risk of major bleeding was found for dabigatran (HR = 0.67, 95% CI: 0.47–0.96). Certainty of evidence was rated as moderate for VTE of all the comparisons due to risk of bias in the included trials. Regarding major bleeding, certainty of evidence was moderate for dabigatran because of risk of bias in included trials, while it was rated as low for rivaroxaban, apixaban, and edoxaban due to risk of bias and inconsistency of results. Dabigatran was related with the lowest risk of CRNB, and rivaroxaban seemed to have a smallest risk of death among the four comparisons between DOACs and non-DOACs.

Table 2 displays the summarized characteristics of trials included in indirect comparisons for assessment of transitivity. In general, all the included trials were comparable. The average ages, sex composition, and percentages of patients with renal dysfunction were highly similar among the RCTs. Their outcome definitions and evaluations kept consistent with each other. When comparison groups were VKA or LMWH plus VKA, the time in therapeutic range in the control arms ranged from 60 to 70%. The treatment administration and duration varied among the four DOACs. Different follow-up periods were also observed for the included RCTs.

**Table 2:** Summarized characteristics of trials included in indirect comparisons for assessment of transitivity^*^.

**Potential effect modifiers**	**Dabigatran**	**Rivaroxaban**	**Apixaban**	**Edoxaban**
Age (years)^a^	55 (55–56)	58.5 (56–71)	60 (56–67)	60 (56–64)
Percentage of female patients^a^	39% (39–42%)	43% (35–53%)	43% (35–52%)	45% (43–47%)
Percentage of patients with renal dysfunction^a^	5.0%^*c*^	8.8% (6.9–21.5%)	6.5% (5.0–9.3%)	6.9% (6.5–7.3%)
Percentage of patients with cancer^a^^,^^b^	4.2% (3.9–5.0%)	7.7% (4.7–12.0%)	7.0% (2.5–9.6%)	9.2%^c^
Treatment administration	150 mg twice daily	15, 20, 30, or 40 mg twice daily; or 10 mg once daily	2.5, 5, or 10 mg twice daily; or 20 mg once daily	30 or 60 mg once daily
Treatment duration (months)^a^	6^c^	6 (1–12)	3 (1–8)	7.5 (3–12)
**Definition and assessment of VTE/MB**	**Consistent between all the trials**			
Follow-up period (months)^a^	6 (6–36)	7.5 (3–24)	6 (3–8)	12 (12–12)
Time in therapeutic range when control groups as VKA or LMWH+VKA^a^	60.0% (56.9–65.3%)	60.2% (57.7–62.7%)	70.1%^c^	63.5%*^c^*

* *Unless specified, data only shown for the intervention groups assuming clinical equipoise between DOAC groups and non-DOAC groups*.

a Data shown as median (range);

b Data excluding trials that had patients with cancer exclusively;

c *Data only shown for a single trial*.

**Figure 4:**
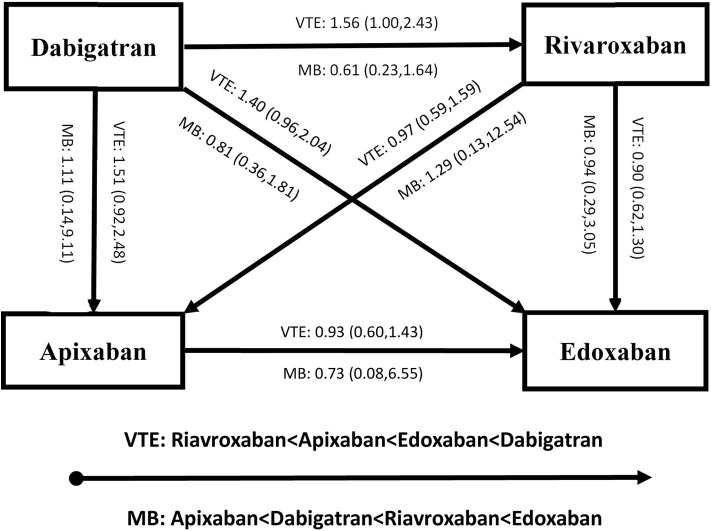
Results of indirect comparison for risk of recurrent VTE and MB (note: arrowhead taken as reference; VTE, venous thromboembolism; MB, major bleeding).

**Figure 5:**
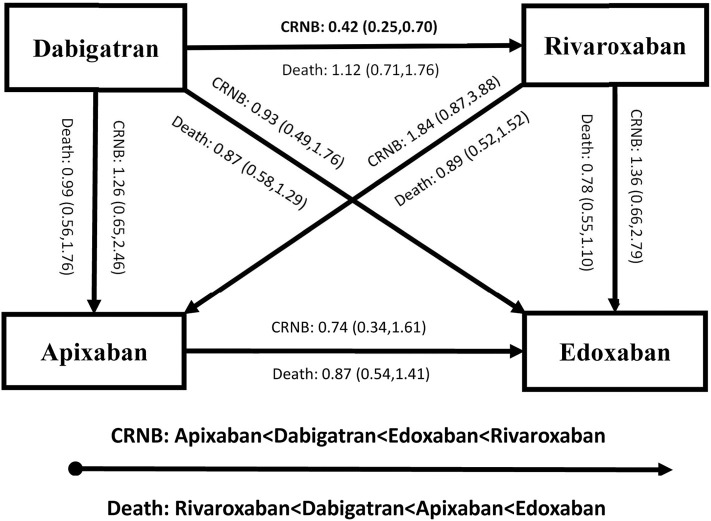
Results of indirect comparison for risk of CRNB and death (note: arrowhead taken as reference; CRNB, clinically relevant non-major bleeding).

Figures 4, 5 present results for the indirect comparison. Figure 4 shows findings from the pairwise comparisons for primary outcomes, where all the results were not statistically significant. Regarding risk of recurrent VTE, rivaroxaban seemed to be the most effective among the four DOACs, followed by apixaban, edoxaban, and dabigatran. Certainty of evidence for VTE was rated as low in all the pairwise indirect comparisons due to concerns for intransitivity and the moderate certainty of evidence in direct comparison. For the safety effect, apixaban was non-significantly related to the lowest risk of major bleeding, followed by dabigatran, rivaroxaban, and edoxaban. All the quality of evidence for major bleeding was rated as very low. Figure 5 displays results for secondary outcomes. Apixaban tended to be associated with the smallest risk of CRNB, while rivaroxaban had the highest CRNB risk. Only the dabigatran-rivaroxaban comparison yielded a significant result for risk of CRNB (HR = 0.42, 95% CI: 0.25–0.70, rivaroxaban taken as reference). As regards death, no significant difference in treatment effect was found among the four DOACs.

Results from the sensitivity analyses were in general consistent with the main findings (Supplemental Table 2). Likewise, subgroup analyses yielded similar results between DVT and PE, and between different types of control groups, in which all the subgroup effect tests were not significant.

## Discussion

In our study using indirect comparison technique, we found no significant difference in treatment effect among DOACs in patients with acute VTE. The quality of evidence was rated as low for VTE and very low for major bleeding in all the indirect comparison among DOACs. The use of a DOAC for acute VTE required more evidence to support decision-making in clinical practice, where head-to-head trials would be ultimately needed to confirm the choice of DOACs.

Current guidelines do not recommend a specific DOAC with a best benefit-harm profile, leaving an important gap in evidence-based decision aid. Cohort studies including retrospective and registry research, could provide direct comparative evidence among the DOACs. Nevertheless, their methodology is limited and challenging to inform an optimal DOAC choice (33). While the choice of a DOAC remained largely dependent on physician and/or patient preferences, expense reimbursement and medication availability, employing evidence from high-quality RCTs to perform an indirect comparison may provide some assistance to the DOAC prescription. In the literature, there had been some published studies based on an indirect comparison approach from RCTs. Two indirect comparison studies focusing on non-cancer patients with acute VTE included six RCTs and reported no difference in treatment effect of DOACs (34, 35). Another study comparing three DOACs (apixaban, rivaroxaban, and edoxaban) with dalteparin also found no significant difference among the DOACs for patients with cancer, based on data from three RCTs (36). Our findings were in line with these previous research. However, we found that dabigatran seemed to have a highest risk of recurrent VTE, but a second lowest risk of major bleeding. Alert being difficult to interpret this phenomenon, part of the reasons may be the different pharmacological effect between dabigatran (thrombin inhibitor) and others (factor Xa inhibitors). Different patient characteristics, various treatment administration and study periods may also play a role in the different effect of dabigatran when compared with the factor Xa inhibitors.

Regarding the assessment of transitivity between the included trials, one of the major differences may exist in the treatment for the control groups in the lead-in phases. While some trials used a heparinoid initially before the random assignment, other trials assigned patients to DOAC or control group immediately after their diagnosis of acute VTE (Table 1). Another potential incomparability may be the blinding of study design between the included studies (Figure 2). However, it remained uncertain about whether and to what extent the lack of blinding or different types of blinding would make an impact on participant performance and outcome assessment. Likewise, little would be known about the influence of blinding on our results from the indirect comparison. Patients with cancer and acute VTE may respond differently from those without cancer when treating with a DOAC (37). Therefore, even though the percentages of patients with cancer were similar in the trials that did not enroll cancer-patients exclusively (Table 2), the subtle difference may yield an unmeasurable impact on the transitivity among the included trials. We ran a sensitivity analysis excluding those trials that had patients with cancer exclusively (SELECT-D, ADAM VTE, and Hokusai VTE Cancer), and observed similar results to main findings (Supplemental Table 2). Nevertheless, it remained difficult to fully assess the assumption of transitivity between the included RCTs in our indirect comparison analysis.

Our study has some limitations. An indirect comparison technique is prone to potential and unquantified bias that could be minimized in head-to-head comparative RCTs (11). The number of included trials was small to restrict our further analyses. While the transitivity assumption could not be firmly explored due to lack of formal tests available, data from the included RCTs indicated that the assumption may be challenged or violated, even though it had been argued that the challenging similarity among the included trials may reflect variations in populations in real-world practice (38). We included all trials investigating efficacy and safety of the DOACs in patients with acute VTE, aiming to provide a comprehensive picture for the comparative treatment effect of DOACs based on all the available data. While heterogeneity was introduced especially regarding populations and controls, subgroup and sensitivity analyses were conducted to mitigate this concern. Some trials did not provide information on HRs; we used the reported RRs (relative risks) for the pooled analyses even given the obvious difference between these two effect measures. A *post-hoc* sensitivity analysis was performed restricting the data of HRs for pooled analyses, yielding the results largely consistent with the main findings. Due to the limited availability of data, no more exploratory analyses could be conducted. For instance, no subgroup analyses by age, sex, history of VTE, treatment administration and duration, or follow-up period could be further performed. The evidence quality of all indirect comparison was low for VTE and very low for major bleeding, reflecting that the current evidence could not provide sufficient information to aid with the selection of a DOAC in clinical practice.

Our results could not provide evidence on favored effects for a specific DOAC; and results from indirect comparison should be interpreted with cautions. However, this study presented the current available evidence from RCTs and highlighted the need for head-to-head comparative trials. While data from the direct comparative trials could not be available in the near future, more methodological research including simulation studies, using a net-benefit or benefit-harm approach, running ranking probability analysis, and developing decision aids with machine-learning may be a worthwhile endeavor to help with the choice of DOACs in patients with acute VTE.

To conclude, no significant difference in the efficacy and safety was found among the DOACs in patients with acute VTE, based on results from the indirect comparison. More evidence from direct comparative trials is needed to further inform the choice of DOACs in patients with acute VTE in clinical practice.

## Data Availability Statement

The original contributions presented in the study are included in the article/Supplementary Materials, further inquiries can be directed to the corresponding author/s.

## Author Contributions

GL and JZe: conceptualization. GL and JZe: data curation; GL, JZe, and JZh: formal analysis. GL: funding acquisition. GL and LT: methodology. GL: supervision. GL and LT: validation. GL and JZe: writing—original draft. JZh and LT: writing—review and editing. All authors: contributed to the article and approved the submitted version.

## Conflict of Interest

The authors declare that the research was conducted in the absence of any commercial or financial relationships that could be construed as a potential conflict of interest.
