# Supplementary material for: Comparative Effects Between Direct Oral Anticoagulants for Acute Venous Thromboembolism: Indirect Comparison From Randomized Controlled Trials
Source: Front Med (Lausanne). 2020 Jun 19;7:280. doi: 10.3389/fmed.2020.00280 (PMC7316891; doi:10.3389/fmed.2020.00280)
Supplement: Supplementary file 1 [file Data_Sheet_1.pdf]

Supplemental Table 1. Ovid search terms modified for MEDLINE, EMBASE and CINAHL (from inception to Dec 8th, 2019)

Search steps	Search terms
1	(dabigatran or BIBR1048 or BIBR-1048 or BIBR 1048).mp.
2	(rivaroxaban or BAY 59 7939 or BAY 59-7939 or BAY 597939 or BAY59-7939 or BAY597939).mp.
3	(apixaban or BMS-562247 or BMS562247 or BMS 562247).mp.
4	(edoxaban or DU-176b or DU176b or DU 176b).mp.
5	(oral factor Xa inhibitor or oral thrombin inhibitor). mp.
6	exp DOAC
7	exp NOAC
8	1 or 2 or 3 or 4 or 5 or 6 or 7
9	(random$ or placebo$ or single blind$ or double blind$ or triple blind$).ti,ab.
10	(trial or intervention). ti.ab.
11	exp trial
12	9 or 10 or 11
13	(venous thromboembolism or pulmonary embolism or deep vein thrombosis). ti.ab.
14	exp venous thromboembolism
15	(bleeding or hemorrhage). ti. ab.
16	13 or 14 or 15
17	8 and 12 and 16

Supplemental Figure 1. Results from meta-analyses for individual DOAC vs non-DOACs based on the included trials

Supplemental Figure 1a- for risk of recurrent VTE

Study or Subgroup	log[Hazard Ratio]	SE	Weight	Hazard Ratio IV, Random, 95% CI
**Dabigatran**				
RE-MEDY (2003)	0.3646	0.3128	4.8%	1.44 [0.78, 2.66]
RE-COVER I (2009)	0.0953	0.2684	6.1%	1.10 [0.65, 1.86]
RE-COVER II (2014)	0.077	0.267	6.1%	1.08 [0.64, 1.82]
**Subtotal (95% CI)**			**17.0%**	**1.17 [0.85, 1.61]**
Heterogeneity: Tau² = 0.00; Chi² = 0.58, df = 2 (P = 0.75); I² = 0%				
Test for overall effect: Z = 0.99 (P = 0.32)				
**Rivaroxaban**				
ODIXa-DVT (2007)	0.6729	1.2183	0.4%	1.96 [0.18, 21.34]
Einstein-DVT Dose-Ranging Study (2008)	-2.0402	0.955	0.6%	0.13 [0.02, 0.84]
SELECT-D (2018)	-0.844	0.4167	3.0%	0.43 [0.19, 0.97]
EINSTEIN-DVT(2010)	-0.3857	0.2221	7.9%	0.68 [0.44, 1.05]
EINSTEIN-PE (2012)	0.1133	0.2046	8.8%	1.12 [0.75, 1.67]
MAGELLAN (2013)	-0.2614	0.1105	15.9%	0.77 [0.62, 0.96]
**Subtotal (95% CI)**			**36.6%**	**0.75 [0.54, 1.02]**
Heterogeneity: Tau² = 0.06; Chi² = 9.66, df = 5 (P = 0.09); I² = 48%				
Test for overall effect: Z = 1.81 (P = 0.07)				
**Apixaban**				
Botticelli DVT dose-ranging study (2008)	-1.1087	1.2234	0.4%	0.33 [0.03, 3.63]
ADAM VTE Trial (2020)	-2.3126	1.0358	0.5%	0.10 [0.01, 0.75]
ADOPT (2014)	-0.1393	0.1728	10.8%	0.87 [0.62, 1.22]
AMPLIFY (2013)	-0.1744	0.1717	10.8%	0.84 [0.60, 1.18]
**Subtotal (95% CI)**			**22.5%**	**0.78 [0.53, 1.14]**
Heterogeneity: Tau² = 0.05; Chi² = 4.86, df = 3 (P = 0.18); I² = 38%				
Test for overall effect: Z = 1.30 (P = 0.19)				
**Edoxaban**				
Hokusai VTE Cancer (2017)	-0.3425	0.1997	9.1%	0.71 [0.48, 1.05]
Hokusai-VTE (2013)	-0.1165	0.1225	14.8%	0.89 [0.70, 1.13]
**Subtotal (95% CI)**			**23.9%**	**0.84 [0.68, 1.03]**
Heterogeneity: Tau² = 0.00; Chi² = 0.93, df = 1 (P = 0.33); I² = 0%				
Test for overall effect: Z = 1.71 (P = 0.09)				

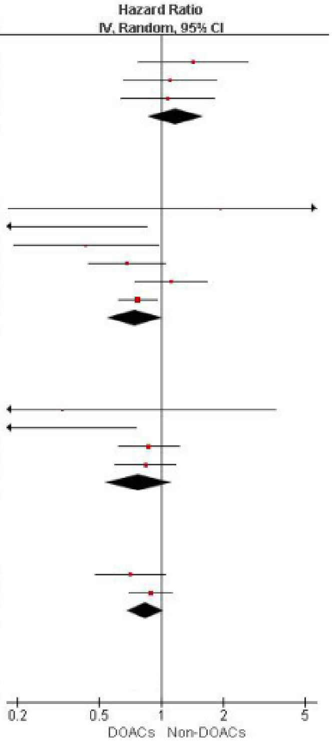

Supplemental Figure 1b- for risk of MB

Study or Subgroup	log[Hazard Ratio]	SE	Weight	Hazard Ratio IV, Random, 95% CI
**Dabigatran**				
RE-COVER I (2009)	-0.1985	0.3062	9.4%	0.82 [0.45, 1.49]
RE-COVER II (2014)	-0.3711	0.3319	9.0%	0.69 [0.36, 1.32]
RE-MEDY (2003)	-0.6539	0.3344	9.0%	0.52 [0.27, 1.00]
**Subtotal (95% CI)**			**27.4%**	**0.67 [0.47, 0.97]**
Heterogeneity: Tau² = 0.00; Chi² = 1.02, df = 2 (P = 0.60); I² = 0%				
Test for overall effect: Z = 2.12 (P = 0.03)				
**Rivaroxaban**				
EINSTEIN-DVT(2010)	-0.4308	0.3459	8.9%	0.65 [0.33, 1.28]
EINSTEIN-PE (2012)	-0.7133	0.2336	10.2%	0.49 [0.31, 0.77]
MAGELLAN (2013)	1.0647	0.3034	9.4%	2.90 [1.60, 5.26]
SELECT-D (2018)	0.6043	0.5051	6.9%	1.83 [0.68, 4.92]
**Subtotal (95% CI)**			**35.4%**	**1.11 [0.44, 2.78]**
Heterogeneity: Tau² = 0.75; Chi² = 24.44, df = 3 (P < 0.0001); I² = 88%				
Test for overall effect: Z = 0.23 (P = 0.82)				
**Apixaban**				
ADOPT (2014)	0.9478	0.4735	7.3%	2.58 [1.02, 6.53]
AMPLIFY (2013)	-1.1712	0.3065	9.4%	0.31 [0.17, 0.57]
**Subtotal (95% CI)**			**16.6%**	**0.87 [0.11, 6.91]**
Heterogeneity: Tau² = 2.09; Chi² = 14.11, df = 1 (P = 0.0002); I² = 93%				
Test for overall effect: Z = 0.13 (P = 0.89)				
**Edoxaban**				
Hokusai VTE Cancer (2017)	0.571	0.2762	9.7%	1.77 [1.03, 3.04]
Hokusai-VTE (2013)	-0.1744	0.1802	10.8%	0.84 [0.59, 1.20]
**Subtotal (95% CI)**			**20.6%**	**1.18 [0.57, 2.45]**
Heterogeneity: Tau² = 0.22; Chi² = 5.11, df = 1 (P = 0.02); I² = 80%				
Test for overall effect: Z = 0.45 (P = 0.65)				

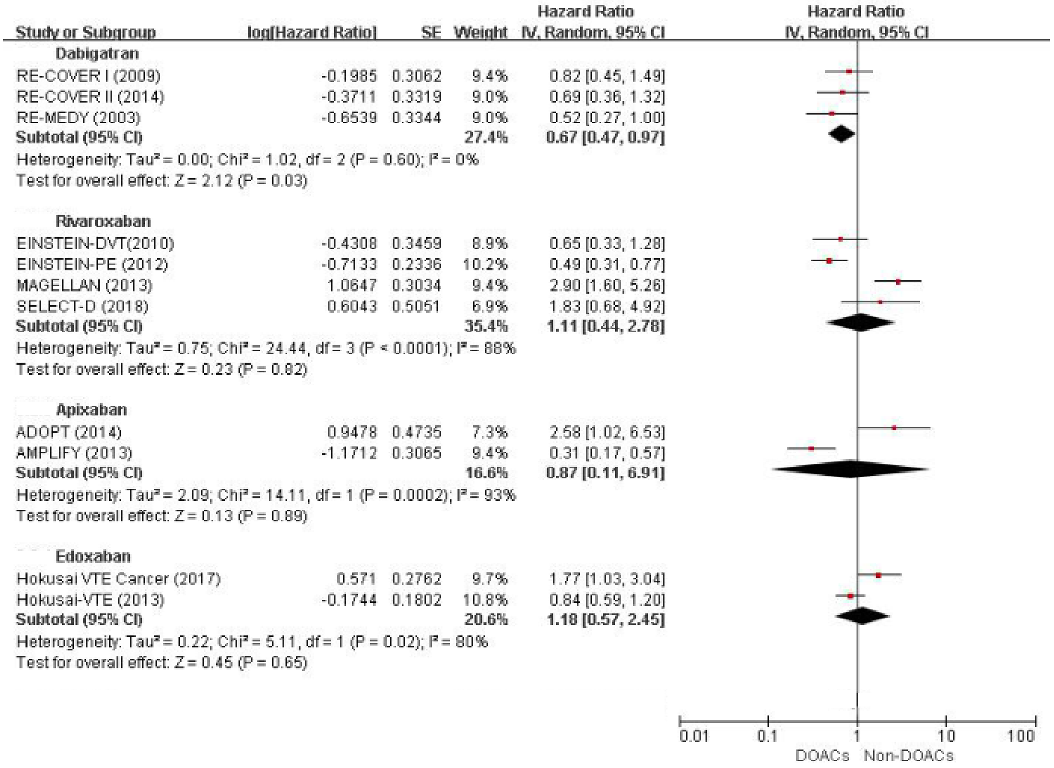

Supplemental Figure 1c- for risk of CRNB

Study or Subgroup	log[Hazard Ratio]	SE	Weight	Hazard Ratio IV, Random, 95% CI
**Dabigatran**				
RE-COVER I (2009)	-0.5447	0.177	7.5%	0.58 [0.41, 0.82]
RE-COVER II (2014)	-0.478	0.1635	7.7%	0.62 [0.45, 0.85]
RE-MEDY (2003)	-0.5798	0.1591	7.7%	0.56 [0.41, 0.76]
**Subtotal (95% CI)**			**22.9%**	**0.59 [0.49, 0.71]**
Heterogeneity: Tau² = 0.00; Chi² = 0.20, df = 2 (P = 0.90); I² = 0%				
Test for overall effect: Z = 5.58 (P < 0.00001)				
**Rivaroxaban**				
Einstein-DVT Dose-Ranging Study (2008)	-1.204	0.6744	2.6%	0.30 [0.08, 1.13]
EINSTEIN-DVT(2010)	0.0488	0.1324	8.0%	1.05 [0.81, 1.36]
EINSTEIN-PE (2012)	-0.0305	0.0983	8.3%	0.97 [0.80, 1.18]
MAGELLAN (2013)	0.9163	0.1536	7.8%	2.50 [1.85, 3.38]
ODIXa-DVT (2007)	0.4447	0.4708	4.1%	1.56 [0.62, 3.93]
SELECT-D (2018)	1.3244	0.4265	4.5%	3.76 [1.63, 8.67]
**Subtotal (95% CI)**			**35.4%**	**1.40 [0.87, 2.24]**
Heterogeneity: Tau² = 0.25; Chi² = 40.08, df = 5 (P < 0.00001); I² = 88%				
Test for overall effect: Z = 1.39 (P = 0.17)				
**Apixaban**				
ADAM VTE Trial (2020)	0.2311	0.5141	3.7%	1.26 [0.46, 3.45]
ADOPT (2014)	0.1484	0.177	7.5%	1.16 [0.82, 1.64]
AMPLIFY (2013)	-0.734	0.1192	8.1%	0.48 [0.38, 0.61]
AMPLIFY-J Study (2015)	-1.1087	0.723	2.4%	0.33 [0.08, 1.36]
Botticelli DVT dose-ranging study (2008)	-0.1054	0.4819	4.0%	0.90 [0.35, 2.31]
**Subtotal (95% CI)**			**25.8%**	**0.76 [0.43, 1.35]**
Heterogeneity: Tau² = 0.28; Chi² = 20.17, df = 4 (P = 0.0005); I² = 80%				
Test for overall effect: Z = 0.94 (P = 0.35)				
**Edoxaban**				
Hokusai VTE Cancer (2017)	0.3221	0.1746	7.5%	1.38 [0.98, 1.94]
Hokusai-VTE (2013)	-0.2231	0.0829	8.4%	0.80 [0.68, 0.94]
**Subtotal (95% CI)**			**16.0%**	**1.03 [0.60, 1.75]**
Heterogeneity: Tau² = 0.13; Chi² = 7.96, df = 1 (P = 0.005); I² = 87%				
Test for overall effect: Z = 0.10 (P = 0.92)				

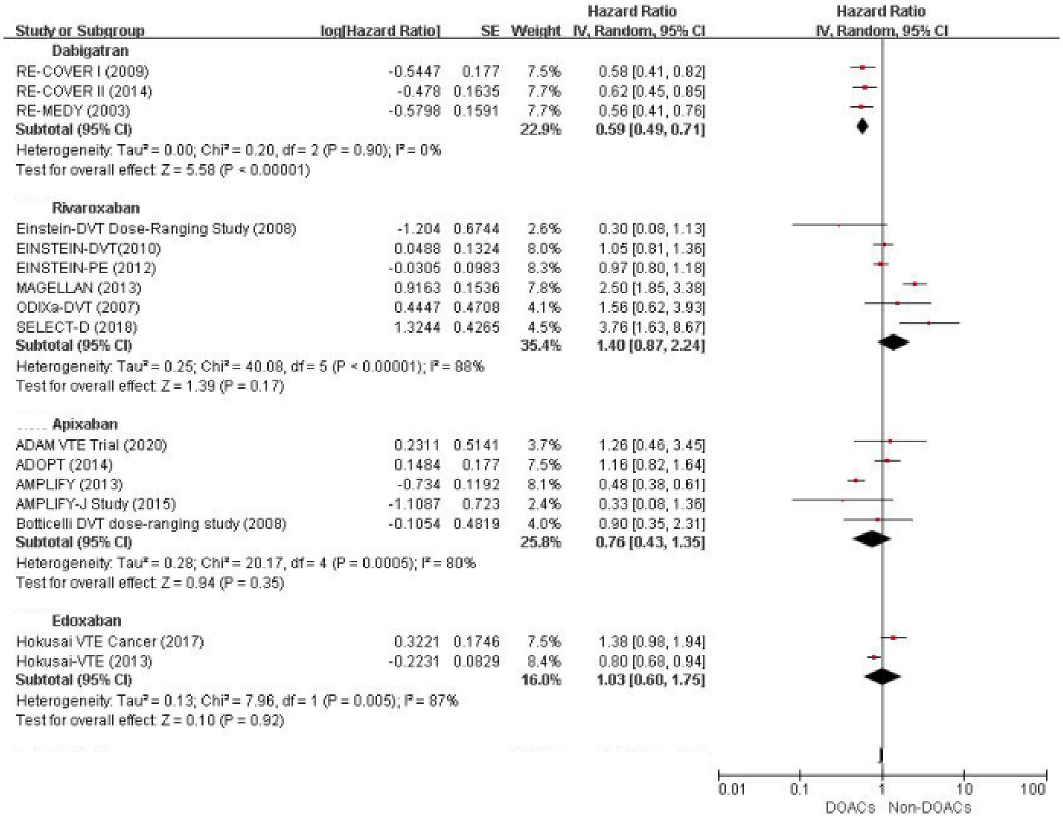

Supplemental Figure 1d- for risk of death

Study or Subgroup	log[Hazard Ratio]	SE	Weight	Hazard Ratio IV, Random, 95% CI
**Dabigatran**				
RE-COVER I (2009)	-0.0202	0.3136	3.4%	0.98 [0.53, 1.81]
RE-COVER II (2014)	-0.0202	0.2855	4.1%	0.98 [0.56, 1.71]
RE-MEDY (2003)	-0.1054	0.3315	3.0%	0.90 [0.47, 1.72]
**Subtotal (95% CI)**			**10.5%**	**0.96 [0.67, 1.36]**
Heterogeneity: Tau² = 0.00; Chi² = 0.05, df = 2 (P = 0.98); I² = 0%				
Test for overall effect: Z = 0.25 (P = 0.80)				
**Rivaroxaban**				
Einstein-DVT Dose-Ranging Study (2008)	-0.9163	0.8212	0.5%	0.40 [0.08, 2.00]
EINSTEIN-DVT(2010)	-0.4005	0.2145	7.2%	0.67 [0.44, 1.02]
EINSTEIN-PE (2012)	0.1222	0.1957	8.7%	1.13 [0.77, 1.66]
MAGELLAN (2013)	0.5247	0.3506	2.7%	1.69 [0.85, 3.36]
SELECT-D (2018)	-0.1508	0.2189	6.9%	0.86 [0.56, 1.32]
**Subtotal (95% CI)**			**26.1%**	**0.94 [0.68, 1.30]**
Heterogeneity: Tau² = 0.06; Chi² = 7.41, df = 4 (P = 0.12); I² = 46%				
Test for overall effect: Z = 0.39 (P = 0.70)				
**Apixaban**				
ADAM VTE Trial (2020)	0.4055	0.3537	2.7%	1.50 [0.75, 3.00]
ADOPT (2014)	-0.3711	0.9369	0.4%	0.69 [0.11, 4.33]
AMPLIFY (2013)	-0.2357	0.2037	8.0%	0.79 [0.53, 1.18]
**Subtotal (95% CI)**			**11.1%**	**0.96 [0.61, 1.50]**
Heterogeneity: Tau² = 0.04; Chi² = 2.56, df = 2 (P = 0.28); I² = 22%				
Test for overall effect: Z = 0.19 (P = 0.85)				
**Edoxaban**				
Hokusai VTE Cancer (2017)	0.1133	0.1004	31.8%	1.12 [0.92, 1.36]
Hokusai-VTE (2013)	0.0488	0.1261	20.5%	1.05 [0.82, 1.34]
**Subtotal (95% CI)**			**52.3%**	**1.09 [0.94, 1.27]**
Heterogeneity: Tau² = 0.00; Chi² = 0.16, df = 1 (P = 0.69); I² = 0%				
Test for overall effect: Z = 1.12 (P = 0.26)				

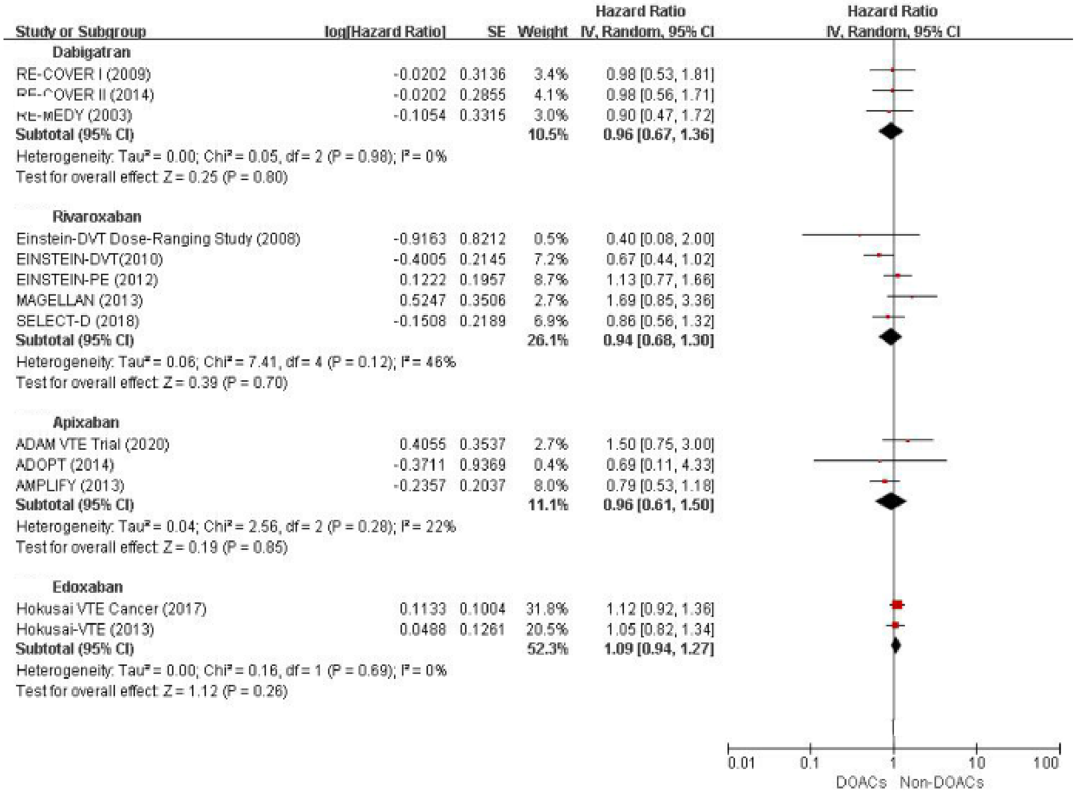

Supplemental Table 2. Results from subgroup and sensitivity analyses for risk of recurrent VTE and MB

Study outcome		D vs R	D vs A	D vs E	R vs A	R vs E	A vs E
**VTE**							
Subgroup analysis							
**Different non-DOACs as controls**	VKAs	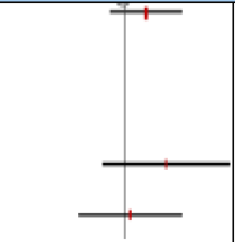	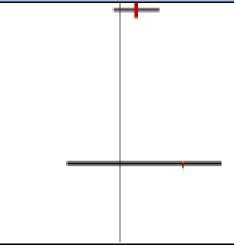	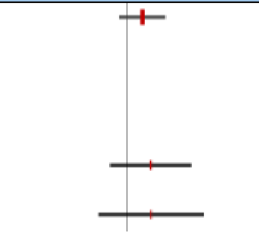	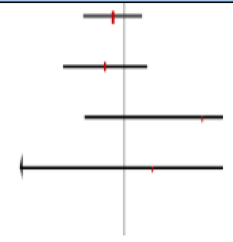	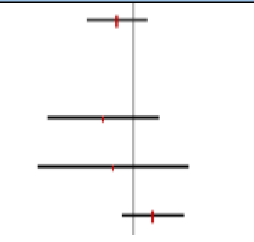	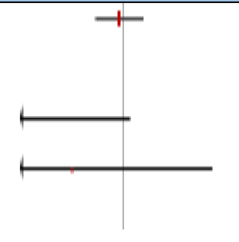
	LMWH						
	UFH						
**Different VTE profile**	DVT						
	PE						
Sensitivity analysis							
Using fixed-effects model		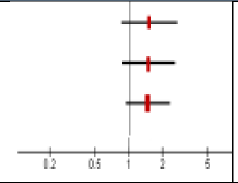	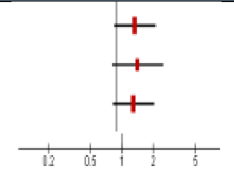	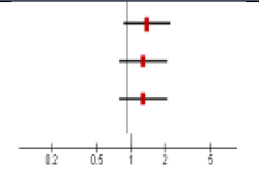	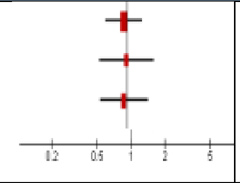	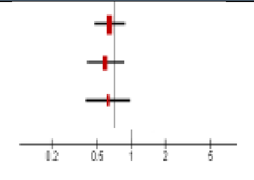	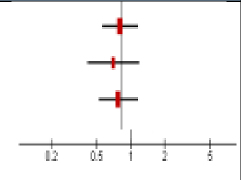
Including low-risk-of bias studies only							
Excluding the trials in which the percentages of patients with cancer were over 10%							
**MB**							
Subgroup analysis							
**Different non-DOACs as controls**	VKAs	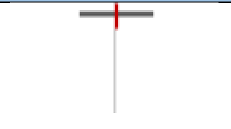	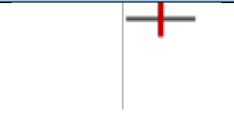	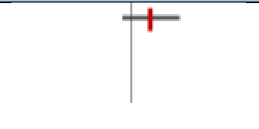	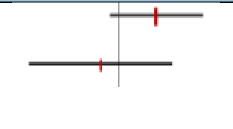	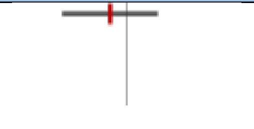	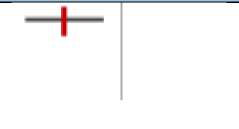
	LMWH						
	UFH						
Sensitivity analysis							
Using fixed-effects model		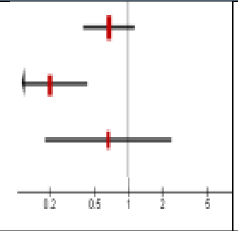	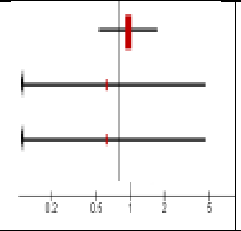	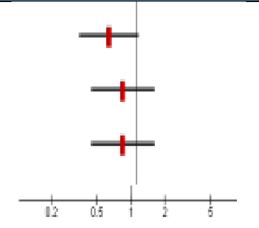	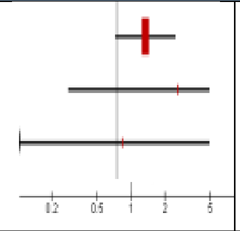	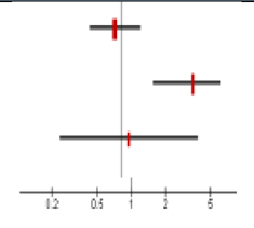	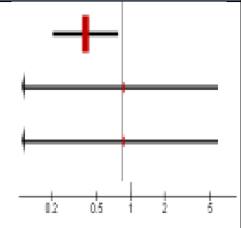
Including low-risk-of bias studies only							
Excluding the trials in which the percentages of patients with cancer were over 10%							

Supplemental Table 3. PRISMA checklist for reporting of this study

Section/topic	#	Checklist item	Reported on page #
**TITLE**			
Title	1	Identify the report as a systematic review, meta-analysis, or both.	1
**ABSTRACT**			
Structured summary	2	Provide a structured summary including, as applicable: background; objectives; data sources; study eligibility criteria, participants, and interventions; study appraisal and synthesis methods; results; limitations; conclusions and implications of key findings; systematic review registration number.	2
**INTRODUCTION**			
Rationale	3	Describe the rationale for the review in the context of what is already known.	3
Objectives	4	Provide an explicit statement of questions being addressed with reference to participants, interventions, comparisons, outcomes, and study design (PICOS).	3
**METHODS**			
Protocol and registration	5	Indicate if a review protocol exists, if and where it can be accessed (e.g., Web address), and, if available, provide registration information including registration number.	NA
Eligibility criteria	6	Specify study characteristics (e.g., PICOS, length of follow-up) and report characteristics (e.g., years considered, language, publication status) used as criteria for eligibility, giving rationale.	3-4
Information sources	7	Describe all information sources (e.g., databases with dates of coverage, contact with study authors to identify additional studies) in the search and date last searched.	3
Search	8	Present full electronic search strategy for at least one database, including any limits used, such that it could be repeated.	3
Study selection	9	State the process for selecting studies (i.e., screening, eligibility, included in systematic review, and, if applicable, included in the meta-analysis).	4
Data collection process	10	Describe method of data extraction from reports (e.g., piloted forms, independently, in duplicate) and any processes for obtaining and confirming data from investigators.	4
Data items	11	List and define all variables for which data were sought (e.g., PICOS, funding sources) and any assumptions and simplifications made.	4
Risk of bias in individual studies	12	Describe methods used for assessing risk of bias of individual studies (including specification of whether this was done at the study or outcome level), and how this information is to be used in any data synthesis.	4

Summary measures	13	State the principal summary measures (e.g., risk ratio, difference in means).	4-5
Synthesis of results	14	Describe the methods of handling data and combining results of studies, if done, including measures of consistency (e.g., I^2) for each meta-analysis.	5

Risk of bias across studies	15	Specify any assessment of risk of bias that may affect the cumulative evidence (e.g., publication bias, selective reporting within studies).	5
Additional analyses	16	Describe methods of additional analyses (e.g., sensitivity or subgroup analyses, meta-regression), if done, indicating which were pre-specified.	5
**RESULTS**			
Study selection	17	Give numbers of studies screened, assessed for eligibility, and included in the review, with reasons for exclusions at each stage, ideally with a flow diagram.	5, Figure 1
Study characteristics	18	For each study, present characteristics for which data were extracted (e.g., study size, PICOS, follow-up period) and provide the citations.	5-6, Table 1, Table 2
Risk of bias within studies	19	Present data on risk of bias of each study and, if available, any outcome level assessment (see item 12).	6, Figure 2
Results of individual studies	20	For all outcomes considered (benefits or harms), present, for each study: (a) simple summary data for each intervention group (b) effect estimates and confidence intervals, ideally with a forest plot.	Figures 3-5
Synthesis of results	21	Present results of each meta-analysis done, including confidence intervals and measures of consistency.	6-7
Risk of bias across studies	22	Present results of any assessment of risk of bias across studies (see Item 15).	6-7
Additional analysis	23	Give results of additional analyses, if done (e.g., sensitivity or subgroup analyses, meta-regression [see Item 16]).	7
**DISCUSSION**			
Summary of evidence	24	Summarize the main findings including the strength of evidence for each main outcome; consider their relevance to key groups (e.g., healthcare providers, users, and policy makers).	7
Limitations	25	Discuss limitations at study and outcome level (e.g., risk of bias), and at review-level (e.g., incomplete retrieval of identified research, reporting bias).	8-9
Conclusions	26	Provide a general interpretation of the results in the context of other evidence, and implications for future research.	9
**FUNDING**			

Funding	27	Describe sources of funding for the systematic review and other support (e.g., supply of data); role of funders for the systematic review.	10
